# Reconstructing radiation strategy: preoperative tumor-bed boost before breast-conserving surgery – innovation or overreach?

**DOI:** 10.1097/JS9.0000000000003228

**Published:** 2025-08-22

**Authors:** Weibin Lian, Debo Chen

**Affiliations:** Department of Breast Surgery, Affiliated Quanzhou First Hospital of Fujian Medical University, Quanzhou, Fujian, P.R. China

*Dear Editor,*

We have carefully studied the phase II clinical trial published by Dong *et al* in your esteemed journal, which investigates an emerging radiotherapy paradigm: delivering a single-fraction 10 Gy tumor-bed boost before breast-conserving surgery (BCS), followed by postoperative ultra-hypofractionated whole-breast irradiation^[1]^. While the feasibility and short-term safety are demonstrated, this approach raises several unsettling oncological, radiobiological, and surgical concerns that warrant deeper scrutiny.

1. Oncological Logic: Are We Fixing a Problem That Doesn’t Exist?

The rationale of “preempting” tumor bed shift post-oncoplastic surgery is intellectually appealing but clinically debatable. Long-established techniques **–** clip placement, seroma-guided imaging, and IORT **–** already provide reliable localization. A robust Cochrane review confirms the benefit of postoperative boost in reducing local recurrence, especially in high-risk groups, without necessitating a timeline reversal^[2]^. The attempt to “solve” a modest planning challenge with an invasive paradigm shift appears disproportionate.

2. Surgical Consequences: Pre-radiated Tissues May Compromise Oncoplastic Flexibility

Delivering 10 Gy preoperatively may adversely affect surgical handling. The fibrosis, vascular compromise, and potential changes in tissue pliability after irradiation are not trivial, particularly in young patients requiring extensive glandular remodeling. The observed 19.4% complication rate **–** including 11.1% Clavien–Dindo grade ≥3 **–** deserves more than passive acknowledgment. Unlike intraoperative boost approaches (e.g., IOERT), which integrate radiation seamlessly during surgery^[3]^, this method preconditions tissue without intraoperative control.

3. Patient-Reported Outcomes: Red Flags in Chest Well-being

The BREAST-Q results in Dong *et al*’s trial revealed significantly lower chest physical well-being scores and more concern about arm swelling in the preoperative boost group. While other domains (e.g., breast satisfaction and psychosocial well-being) did not differ, this decline in physical chest comfort may hint at early tissue reaction, surgical recovery delay, or even lymphatic dysfunction. Prior studies suggest that radiation delivered close to axillary levels **–** particularly without surgical insight **–** can exacerbate soft tissue stiffness and subclinical lymphedema, affecting quality of life even in the absence of gross complications^[4]^.

These subtle impairments may become clinically significant with longer follow-up, particularly in younger patients who prioritize esthetics and arm function. Therefore, early negative shifts in patient-reported outcomes **–** even if not statistically dramatic **–** deserve heightened attention.

4. Lack of Long-Term Control and Comparative Oncologic Efficacy

No recurrence at 9.8 months is insufficient to infer oncological safety. Other strategies like intraoperative tumor-bed boost during BCS (TARGIT-IORT) have shown non-inferiority or even trends toward improved survival after neoadjuvant therapy^[5]^. Importantly, these avoid unnecessary radiation exposure to unresected tissue. What if the patient has positive margins or is upstaged after final pathology? The prior radiation may render reoperation or mastectomy more complex.

5. Radiobiological Discrepancy: Fragmented Dose Logic

While the ultra-hypofractionated WBI (26 Gy in five fractions) is supported by the FAST-Forward trial logic, the isolated 10 Gy boost lacks comparative validation in the preoperative setting. In fact, most boost regimens assume the tumor has been removed and only microscopic disease remains. A 10 Gy single dose to gross disease pre-resection might not offer radiobiologic advantage and could introduce inflammatory distortion, obscuring margins and increasing surgical uncertainty.

In conclusion, while innovation is essential, preoperative tumor bed boost radiotherapy must not outpace its evidence base. Prudence, not premature adoption, should guide clinical implementation^[6]^.

## Data Availability

Data sharing is not applicable to this article as no datasets were generated or analyzed during the current study.
